# Epidemiological characteristics of methicillin-resistant *Staphylococcus aureus* isolates from bloodstream cultures at University Hospital in the Czech Republic

**DOI:** 10.1007/s12223-020-00782-9

**Published:** 2020-03-14

**Authors:** Katerina Neradova, Marta Fridrichova, Vladislav Jakubu, Katarina Pomorska, Helena Zemlickova

**Affiliations:** 1 Department of Clinical Microbiology, Faculty of Medicine and University Hospital, Charles University, Hradec Kralove, Czech Republic; 2 Department of Medical Microbiology, Charles University, 3rd Faculty of Medicine, Prague, Czech Republic; 3 Faculty of Medicine in Hradec Kralove, Charles University, Hradec Kralove, Czech Republic; 4 National Reference Laboratory for Antibiotics, National Institute of Public Health, Prague, Czech Republic

## Abstract

The aim of this study was to trace the dynamic changes of methicillin-resistant *Staphylococcus aureus* (MRSA) lineages in the local hospital in both the national and international context. We describe genotypic and phenotypic characterization of 62 non-duplicate MRSA isolates collected during 2010–2016 at University Hospital in Hradec Kralove, Czech Republic. The isolates were characterized by multilocus sequence typing (MLST), *spa* typing, and staphylococcal cassette chromosome *mec* typing (SCC*mec* typing). Eight different genotypes were described; ST225-t003-II (32/62, 52%), ST5-t002-II (13/62, 22%), and ST225-t014-II (12/62, 21%) were constantly detected over the 7-year follow-up period. The genotypes ST225-t151-II, ST225-t1282-II, ST225-t1623-II, ST78-t2832-II, and ST225-t8799-II occurred only once in the period reported. The majority of the strains, represented by ST225, belonged to clonal complex 5 (CC5).

## Introduction

Methicillin-resistant *Staphylococcus aureus* (MRSA) is an important human pathogen causing a wide range of infectious diseases, from local skin and soft tissue lesions to serious invasive infections (Palavecino 2020). MRSA bloodstream infections (BSI) are associated with higher mortality rates and worse outcome than bloodstream infections due to methicillin-susceptible *S. aureus* (MSSA) (de Kraker et al. 2011). Hospital-acquired MRSA (HA-MRSA) is a typical nosocomial pathogen, but in recent decades, strains with different epidemiological and genotypical characteristics have been described. These community-associated MRSA strains are spreading in younger people without previous contact with the healthcare system (Naimi et al. 2003). Clonal replacement of predominant HA-MRSA strains has occurred in the Czech Republic several times during recent decades, as shown by previous multilocus sequence typing (MLST) studies (Melter et al. 1999; Melter et al. 2003; Melter et al. 2006; Grundmann et al. 2010). The introduction of CC5 in 2006/2007 was accompanied by an increasing percentage of MRSA from BSI (Grundmann et al. 2010). The prevalence rate of MRSA in BSI increased from 6.1% in 2003 to 12.9% in 2005 and predominated till 2007 when the dominance of CC5 among isolates from BSI was documented for the first time in the Czech Republic (Grundmann et al. 2010). This situation has remained stable for a decade, currently oscillating around 13% (± 2%). According to the European Antimicrobial Resistance Surveillance Network (EARS-Net), the prevalence of MRSA among BSI isolates in the Czech Republic is close to the European population-weighted mean (European Centre for Disease Prevention and Control Antimicrobial resistance surveillance in Europe 2016). Since 2007, a nationwide study of MRSA epidemiology has been lacking, the latest information being provided by a study from 2014 describing a local CC5 MRSA outbreak infection in an Intensive Care Unit (ICU) (Stock et al. 2016).

Not only is the description of the present dominant clones epidemiologically significant, but also the newly emerging sporadic clones can be a reservoir for epidemic clones in the future. For targeted preventive measures (e.g., screening for carriers, isolation of positive cases), knowledge about the clonal epidemiology of the local MRSA population is essential. The aim of this study was to trace dynamic changes of nosocomial lineages in the University Hospital and investigate the dominant MRSA genotypes causing bloodstream infections.

## Material and methods

### Hospital

University Hospital Hradec Kralove is a university hospital with 1375-bed capacity (University Hospital Hradec Kralove Annual Report 2017).

### Case definition

A bloodstream infection is defined as one or more positive blood cultures associated with systemic signs of infection, such as fevers, chills, and/or hypotension (Suetens et al. 2015).

### Epidemiological data

Epidemiological data on the duration of hospitalization, MRSA colonization, or site of possible infection were obtained by retrospective analysis of patient records (FONS Openlims, STAPRO s.r.o.; ver. 5.35.51.01) and records of Department of Hospital Epidemiology and Infection Control. MRSA colonization screening was performed in indicated cases repeatedly from suitable locations by swabbing with a cotton swab, which was transported to the laboratory in the transport medium (Copan Transystem®). The swabs were cultured for 18 h (Oxoid™ Columbia Blood Agar Base, Thermo Scientific™ and chromogenic agar MRSA Select™, Bio-Rad), and identification was performed with matrix-assisted laser desorption/ionization time of flight mass spectrometry (MALDI-TOF MS, Bruker Microflex LT™, Bruker Daltonics).

### Bacterial strains

If a BSI is suspected, at least 10 ml of blood is taken into each blood culture bottle (BD BACTECTM Plus Aerobic/F Medium, BD BACTECTM Lytic/10 Anaerobic/F Medium). It is recommended to take at least 2–3 sets of bottles, which are immediately sent to the laboratory for further processing. Blood-culture takes place in the automatic system for 5 days (Becton Dickinson BACTEC 9240 Blood Culture System). When growth of the bacteria is detected, the microorganism is cultured (Oxoid™ Columbia Blood Agar Base, MacConkey agar Oxoid™) and identified. During 2010–2016, 71 MRSA BSI episodes were detected, from which 62 MRSA isolates were collected and further analyzed (Table 1). *S. aureus* was identified morphologically, and identification was confirmed by a positive latex slide agglutination test (Staphytect Plus, Oxoid™), and since 2014 by MALDI-TOF MS. Resistance to methicillin was identified by cefoxitin screen test (EUCAST 2019) and confirmed by detection of *mec*A and *mec*C genes by PCR (Stegger et al. 2012). Only one MRSA strain was enrolled per patient, which was the first isolated in a relevant year.

**Table 1:** *S. aureus* in 2010–2016 at University Hospital Hradec Kralove

Year		2010–2016	2010	2011	2012	2013	2014	2015	2016
Number of hospitalized patients		288,491	41,574	40,515	41,382	40,957	41,766	41,465	40,832
*S. aureus* BSI		521	77	76	71	64	72	71	90
*S. aureus* BSI incidence %		0.2	0.2	0.2	0.2	0.2	0.2	0.2	0.2
MRSA BSI		71	17	12	10	9	6	10	7
MRSA BSI analyzed in this study		62	11	12	10	9	6	10	4
MRSA BSI incidence %		0.02	0.04	0.03	0.02	0.02	0.01	0.02	0.02
MRSA BSI prevalence %		13.6	22	15	12	14	8	14	7
MRSA *spa* type (%)	t003	32 (52)	5 (46)	7 (59)	5 (50)	8 (89)	2 (40)	2 (20)	3 (75)
	t002	13 (22)	4 (36)	1 (8)	2 (20)	0	3 (50)	3 (30)	0
	t014	12 (21)	2 (18)	3 (25)	2 (20)	1 (11)	0	3 (30)	1 (15)
	t151	1 (1)	0	0	0	0	0	1 (10)	0
	t1623	1 (1)	0	0	1 (10)	0	0	0	0
	t2821	1 (1)	0	1 (8)	0	0	0	0	0
	t2832	1 (1)	0	0	0	0	1 (10)	0	0
	t8799	1 (1)	0	0	0	0	0	1 (10)	0

### Antibiotic susceptibility testing

Minimum inhibitory concentrations (MIC) for erythromycin, clindamycin, linezolid, chloramphenicol, tetracycline, ciprofloxacin, trimethoprim/sulfamethoxazole, gentamicin, and vancomycin were determined by broth microdilution method. Data were interpreted according to the criteria of the European Committee on Antimicrobial Susceptibility Testing (EUCAST 2019, www.eucast.org).

### *spa* typing and based upon repeat pattern (BURP) analysis

The typing was performed with primers 1095F (5′-AGACGATCCTTCGGTGAGC-3′) and 1517R (5′-GCTTTTGCAATGTCATTTACTG-3′) (Harmsen et al. 2003). The software Ridom StaphType™ (ver. 2.2.1; Ridom GmbH) was used for sequence and BURP analysis.

### MLST and eBURST

The seven housekeeping genes were used for MLST analysis: carbamate kinase (*arcC*), shikimate dehydrogenase (*aroE*), glycerol kinase (*glp*), guanylate kinase (*gmk*), phosphate acetyltransferase (*pta*), triosephosphate isomerase (*tpi*), and acetyl coenzyme A acetyltransferase (*yqiL*). The fragments were amplified using the primers shown in the reference (Enright et al. 2000). The resulting sequence types (STs) were assigned using software BioNumerics (ver.7.0; Applied Maths). STs were clustered into related groups using the eBURST algorithm. The obtained MLST data were used to derive the corresponding clonal complexes (CCs).

### SCCmec typing

The SCC*mec* types were identified using multiplex PCR based on identification of specific genes within the J regions of particular cassettes (I to V) as described previously (Milheirico et al. 2007).

The incidence was calculated per 100 000 patients, as a number of new cases diseased with MRSA BSI/number of hospitalized patients. The prevalence of MRSA BSI among *S. aureus* BSI was calculated as the number of existing diseases in the time period/number of persons at risk.

## Results

The median age of patients was 68 years (range 1 to 88). Patients diseased with MRSA bloodstream infection were more often men (43/71, 60%); however, the design of the study precludes the drawing of any conclusion as to whether the frequency of MRSA BSI was higher in men.

In 28/62 patients (45%), MRSA colonization preceded the development of BSI. The common colonization sites in our study group were surgical or chronic wounds (9/62, 15%), followed by the respiratory tract (5/62, 8%), skin (4/62, 7%), throat, urine, and nose (each 3/62, 6%). Each patient was screened circa three times during hospitalization. In 34/62 cases (55%), the results of MRSA screening were not known or negative until invasive infection occurred. It took on average 24 days until an initially MRSA-negative inpatient was colonized. This transmission during hospital stay occurred in 11/62 (18%). Their colonization was confirmed by a screening smear, and invasive infection developed later during the hospitalization. Seven of them were colonized by MRSA by direct contact with an unknown carrier. Contact during a stay in the same hospital room caused colonization of skin and mucous membranes in 5 cases. BSI developed during the course of hospitalization. In two cases, a central venous catheter was colonized. That cases were only sporadic, and scattered over time during the study period. These retrospective data were obtained from the study of patients’ records, and epidemiological investigation by molecular typing methods was not the subject of the work and was not performed. The remaining patients 17/62 (27%) were known to be MRSA carriers at the beginning of hospitalization and the source of BSI was probably endogenous. This fact was either already known from past medical history or from confirmatory screening carried out on admission. In many cases, MRSA BSI were catheter-related infections (21/62, 34%), and in 18/62 cases (29%), they were associated with skin and soft tissue infection (SSTI) or osteomyelitis. Pneumonia was present in 5/62 patients (8%); in 4/62 cases (6%) infective endocarditis was diagnosed, and in 14/62 (23%), the source remained unknown (Fig. 2).

**Fig. 1:**
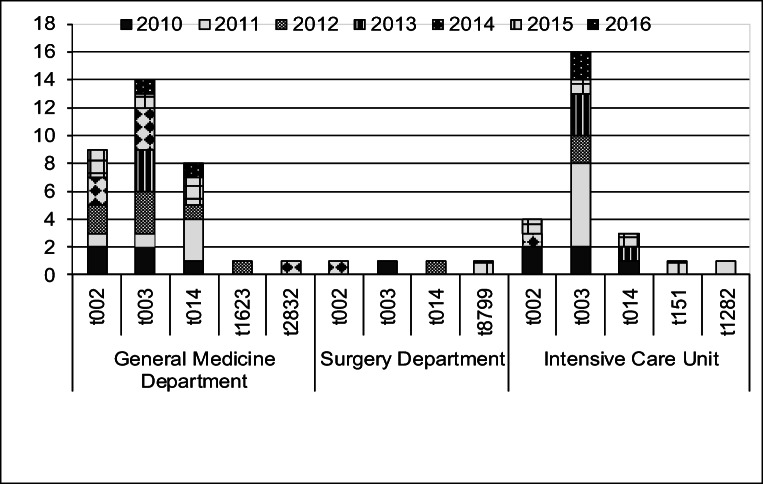
Epidemiological data: occurrence of *spa* types on individual wards of the hospital during the monitored period

BSIs were reported more often from the General Medicine Departments, than from ICUs, and on the surgical wards, they were rare (Fig. 1). The overall incidence rate of *S. aureus* BSI over the 7-year follow-up period was 0.2%; the incidence of MRSA BSI (per 100,000 patients) was 0.02% (Table 1).

Since 2010, the Antibiotic Centre at University Hospital Hradec Kralove has been providing a local annual review of antibiotic resistance. At the beginning of monitoring in 2010, the proportion of MRSA in BSI caused by *S. aureus* was at its highest, 18% (17 MRSA isolates among 77 *S. aureus* isolates). In the following years, there was a gradual decline, to 8% in 2016 (7 MRSA isolates among 90 *S. aureus* isolates).

**Fig. 2:**
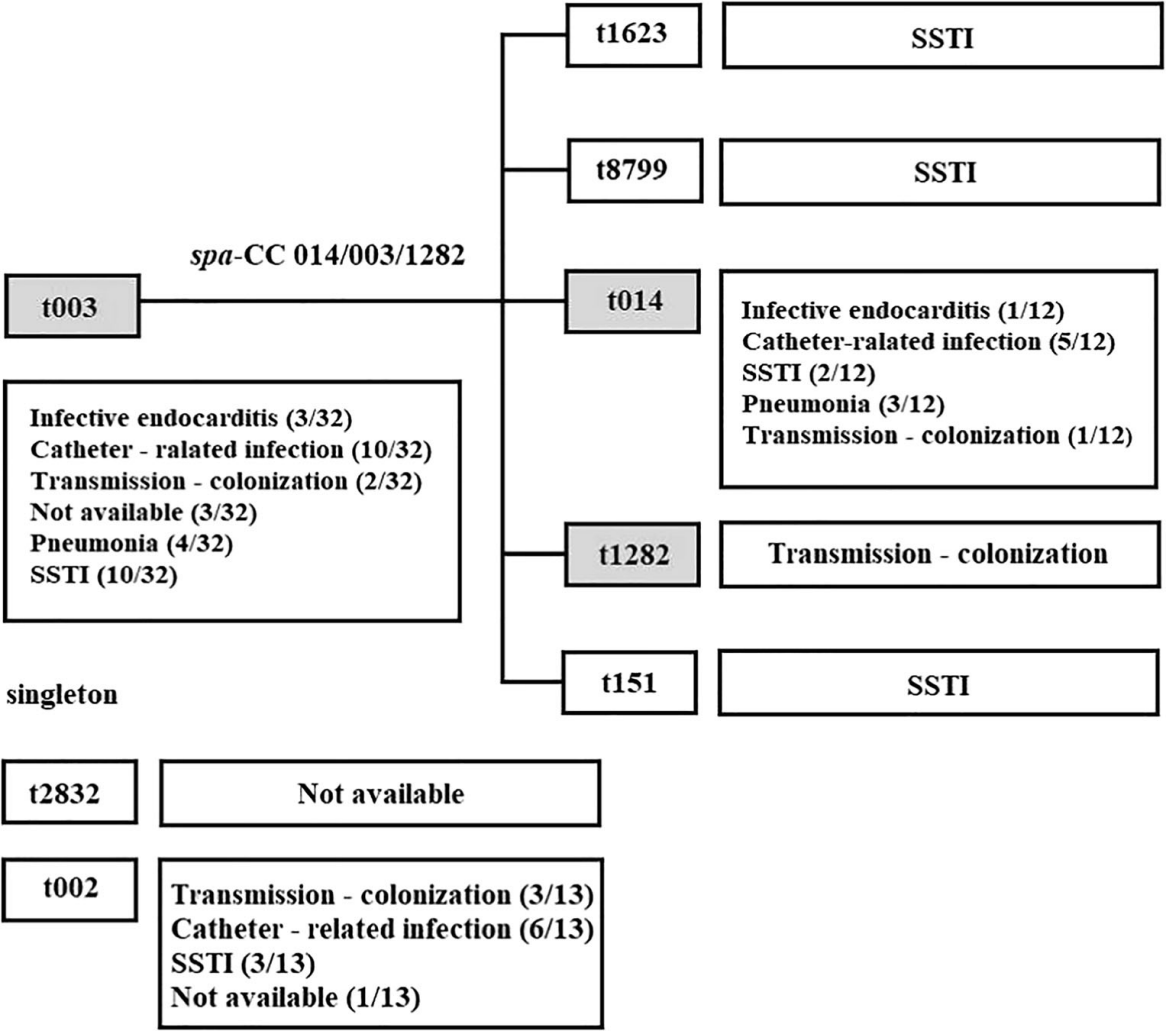
According to BURP analysis, isolates were clustered into relations within *spa*-CC 014/003/1282. *spa* type t003 was determined as the founder, with an evolutionary relationship to t014, t1282, t1623, t151, and t8799. Two singletons were described with no relation to the others: s*pa* type t002 corresponded with ST5 and t2832. The source of BSI is in the table next to the relevant *spa* type

All isolates carried the *mec*A gene and were SCC*mec* type II. The majority of the isolates belonged to CC5 (61/62, 98%), represented by ST225 (48/62, 77%) and ST5 (13/62, 21%). Only one non-CC5 strain was described (Table 2). The ST5 is highly related to ST225 as it is a single locus variant at the *tpi* locus (Table 2). MRSA isolates were classified in 8 distinct *spa* types. Five of them (t151, t1282, t1623, t2832, t8799) were represented in only a single isolate, but the majority of the strains (57/62, 92%) were assigned to three *spa* types (t003, t002, t014). The ST225 included 6 *spa* types (t003, t014, t151, t1282, t1623, t8799). According to BURP analysis, they were clustered into relations within *spa*-CC 014/003/1282. *spa* type t003 was determined as the founder, with an evolutionary relationship to t014, t1282, t1623, t151, and t8799. *spa* type t003 dominated constantly throughout the study period, although there was a small decrease in 2014–2015, when *spa* type t002 slightly prevailed. Two singletons were described with no relation to the others: s*pa* type t002 corresponded with ST5, and *spa* type t2832 corresponded with ST78 (1/62, 2%) (Fig. 2).

**Table 2:** Invasive MRSA strain characteristics

Clonal complex (CC)	*spa* type	Repeat succession	MLST type	Allelic profile^a)^	SCC*mec* type	*mec* type	Total no. of strains (%)	*spa* clonal cluster	Antibiotic resistance^b)^
CC 5 (*n* = 61)	t002	26–23–17–34–17–20–17–12–17–16	ST5	1–4–1–4–12–1–10	II	*mecA*	13 (22)	Singleton	ERY, CLI, CIP, GEN
	t003	26–17–20–17–12–17–17–16	ST225	1–4–1–4–12–25–10	II	*mecA*	32 (52)	CC 014/003/1282	ERY, CLI, CIP, GEN
	t014	26–17–20–17–12–17–17–17–16	ST225	1–4–1–4–12–25–10	II	*mecA*	12 (21)	CC014/003/1282	ERY, CLI, CIP, GEN
	t151	26–17–20–17–16	ST225	1–4–1–4–12–25–10	II	*mecA*	1 (1)	CC014/003/1282	ERY, CLI, CIP
	t1282	26–17–20–17–12–17–17–17–17–16	ST225	1–4–1–4–12–25–10	II	*mecA*	1 (1)	CC014/003/1282	ERY, CLI, CIP
	t1623	07–17–20–17–12–17–17–16	ST225	1–4–1–4–12–25–10	II	*mecA*	1 (1)	CC014/003/1282	ERY, CLI, CIP
	t8799	26–17–20–17–12–17–17–17–17–17–16	ST225	1–4–1–4–12–25–10	II	*mecA*	1 (1)	CC014/003/1282	ERY, CLI, CIP
Non-CC 5 (*n* = 1)	t2832	07–13–13–33–34	ST78	22–1–14–23–12–53	II	*mecA*	1 (1)	Singleton	ERY, CLI

^a)^Internal fragments of seven housekeeping genes: *arcC* (carbamate kinase), *aroE* (shikimate dehydrogenase), *glp* (glycerol kinase), *gmk* (guanylate kinase), *pta* (phosphate acetyltransferase), *tpi* (triosephosphate isomerase), *yqiL* (acetyl coenzyme A acetyltransferase)

^b)^Erythromycin (ERY), clindamycin (CLI), gentamicin (GEN), ciprofloxacin (CIP)

In addition to β-lactams, all isolates of ST5 and ST225 were resistant to erythromycin, clindamycin, and ciprofloxacin, and some strains showed resistance also to gentamicin (5/62, 8%). By contrast, the single isolate of ST78 was resistant only to erythromycin and clindamycin (Table 2).

## Discussion

The average length of hospitalization until invasive MRSA infection developed was 24 days. The average length of hospitalization in the Czech Republic is 6 days (Institute of Health Information and Statistics of the Czech Republic 2018), and so these were especially complicated, long-term hospitalized patients,

According to several studies, MRSA-colonized patients are at fourfold higher risk for invasive MRSA infection than non-colonized patients (Coello et al. 1997). Surgical or chronic wounds (9/62, 15%) were the most commonly colonized in our study group, whereas nasal colonization was not frequent (3/62, 6%). Within Europe the community nasal carriage of MRSA is around 1%, being in Germany 0.7–1.3% (Mehraj et al. 2017; Köck et al. 2016), in Great Britain 1.9% (Gamblin et al. 2013), in France 1.02% (Ficca et al. 2006), and in Italy 0.12% (Zanelli et al. 2002). Carriage prevalence is higher in patients with a history of previous hospitalization up to 10–15% (Riedel et al. 2008, von Eiff et al. 2001). Such a population was characteristic of patients from the group we followed in our study.

The 62 MRSA strains were characterized with sequence type, *spa* type, and SCC*mec* type into 8 different genotypes including ST225-t003-II (32/62, 52%), ST5-t002-II (13/62, 22%), ST225-t014-II (12/62, 21%), and the singly-occurring ST225-t151-II, ST225-t1282-II, ST225-t1623-II, ST78-t2832-II, and ST225-t8799-II, all of suspected with nosocomial origin. The majority of HA-MRSA strains currently circulating worldwide belong to five pandemic lineages, including CC5, CC8, CC22, CC30, and CC45 (Monecke et al. 2011). The three dominant *spa* types in our hospital (t003, t002, t014) belong to the CC5 clade (Rhine-Hesse epidemic MRSA, EMRSA), well documented in central Europe (Grundmann et al. 2010; Sassmannshausen et al. 2016). They also circulate in other Czech hospitals, indicative of an unwelcome spreading and transmission of these clones across the country (Tkadlec et al. 2019). ST225-t003-II is a typical nosocomial strain and has the ability to spread rapidly within a hospital setting. A predominant regional distribution has been described also in hospitals in Germany and its Dutch border area (Sassmannshausen et al. 2016) and in Poland (Grundmann et al. 2010). Special attention should be given to this prevalence, as among the CC5 the emergence of vancomycin resistance has been repeatedly described (Challagundla et al. 2018). To date, 13 types of SCC*mec* types have been reported, labeled I to XIII (Baig et al. 2018). All strains in our collection carried the SCC*mec* type II. Types II and III have frequently been demonstrated in multiresistant HA-MRSA strains (Naimi et al. 2003).

The occurrence of MRSA clones in our country is not constant, and dynamic changes have been reported over the years. Since the beginning of monitoring in 1996–1997, there have been two multiresistant clones widespread in the Czech Republic. The prevailing Brazilian/Hungarian clone ST239-III, described in Portugal, and the Iberian clone ST247-IA, originated from Spain (Melter et al. 1999). In 1999, the prevalent clone was replaced by a variant ST239-IIIA, which differed from the original with the absence of an integrated plasmid p*T181* (Monecke et al. 2011)*.* The Iberian clone maintained its frequency (Melter et al. 2003). After 2001, the epidemic strain ST22-IV, known as EMRSA-15, expanded nationwide. Since 2006/2007, we have witnessed the dominance in the Czech Republic of ST225-t003-II (34/51, 66%), a strain also common in Germany and Poland (Grundmann et al. 2010). The current epidemiological situation in University Hospital Hradec Kralove corresponds to the situation described in 2014 when a local outbreak was described in the ICU of Prague Hospital. The closely related strains ST225-t003-II and ST225-t014-II clearly dominated. During the investigation of local epidemics in 2014, the genetically related strain ST225-t1282-II was also described in two cases. This, together with our present finding, suggests a possible circulation of this clone in Czech hospitals (Stock et al. 2016). ST22-t032-IV (13.7%) was predominantly found in the UK and Ireland, and ST5-t002-II (7.8%) was widespread in Mediterranean regions (Grundmann et al. 2010). Rarely occurring ST225-t151-II seems to be a locally spread lineage reported from Germany (Köck et al. 2009; Cuny et al. 2015), Poland (Kasprzyk et al. 2015) and Belgium (Laurent et al. 2010).

Most MRSA BSIs were reported from General Medicine Departments (33/62), with ST225-t003-II (14/62) slightly prevailing during the observed time period, but with findings of ST5-t002-II (9/60) and ST225-t014-II (8/62) also frequent. In the ICU (27/62), ST225-t003-II (18/27) clearly prevailed. The cases seem to be sporadic; no outbreak having been recorded during the observed time period, and hygienic preventive measures could be considered to have been adequate.

Our results confirm that the clonal population of MRSA in the local University Hospital is homogeneous, and a limited number of dominant clones are spreading among individual wards. Screening for MRSA carriage is still not a routine examination for patients transferred between local facilities, but the risk of transmission should be considered. Some MRSA clones are spread over considerable distances, whereas others are limited to a certain country or even only to a local hospital. Over the years, there have been changes in the dominant clone. This phenomenon has also been described in many European countries (Zarfel et al. 2016).

## Conclusion

The present study confirmed the predominance of CC5 among invasive strains in University Hospital Hradec Kralove. Three closely related genotypes, ST225-t003-II, ST5-t002-II, and ST225-t014-II were the most frequent. They accounted for 92% of tested strains and can be considered endemic at our hospital. Another 5 *spa* types occurred rarely (together 8%) and were considered to be sporadic strains, the close relation to the major *spa* types indicating that these strains could have emerged from dominant *spa* types. The situation in the local hospital reflects the epidemiological situation in the whole country. We are witnessing decreasing incidence of invasive MRSA infections in our hospital, these being sporadic cases during the observed period, and no MRSA outbreak has been recorded. The MRSA BSI rate in the whole country has remained stable for the past few years. This confirms the need for surveillance, typing, and constant reassessment of existing preventive measures to preserve this trend in the future.
